# Full-Field Strain Measurements of the Muscle-Tendon Junction Using X-ray Computed Tomography and Digital Volume Correlation

**DOI:** 10.3390/bioengineering11020162

**Published:** 2024-02-06

**Authors:** Nodoka Iwasaki, Aikaterina Karali, Marta Roldo, Gordon Blunn

**Affiliations:** 1 School of Pharmacy and Biomedical Sciences, University of Portsmouth, Portsmouth PO1 2DT, UK; marta.roldo@port.ac.uk (M.R.); gordon.blunn@port.ac.uk (G.B.); 2 School of Mechanical and Design Engineering, University of Portsmouth, Portsmouth PO1 3DJ, UK; katerina.karali@port.ac.uk

**Keywords:** strain, 3D strain measurement, muscle–tendon junction (MTJ), X-ray computed tomography (XCT), digital volume correlation (DVC), in situ mechanical testing

## Abstract

We report, for the first time, the full-field 3D strain distribution of the muscle-tendon junction (MTJ). Understanding the strain distribution at the junction is crucial for the treatment of injuries and to predict tear formation at this location. Three-dimensional full-field strain distribution of mouse MTJ was measured using X-ray computer tomography (XCT) combined with digital volume correlation (DVC) with the aim of understanding the mechanical behavior of the junction under tensile loading. The interface between the Achilles tendon and the gastrocnemius muscle was harvested from adult mice and stained using 1% phosphotungstic acid in 70% ethanol. In situ XCT combined with DVC was used to image and compute strain distribution at the MTJ under a tensile load (2.4 N). High strain measuring 120,000 µε, 160,000 µε, and 120,000 µε for the first principal stain (ε_p1_), shear strain (γ), and von Mises strain (ε_VM_), respectively, was measured at the MTJ and these values reduced into the body of the muscle or into the tendon. Strain is concentrated at the MTJ, which is at risk of being damaged in activities associated with excessive physical activity.

## 1. Introduction

The muscle-tendon junction (MTJ) is a specialized interface, which transmits the force from the muscle to the tendon [1]. Injuries to the junction between the Achilles tendon and muscle usually occur during sport activities and are due to excessive strain [2] or failure associated with surgery to repair these tissues [3]. Approximately 44,000 patients per year in the US require Achilles-related surgery, with the number predicted to increase due to the aging population [4]. It has been reported that 28% of injuries in the muscle-tendon-bone unit occur at the MTJ [5] and, therefore, this represents a significant problem. Injuries at the MTJ limit the ability of the patients to move; therefore, these injuries are important and affect the quality of life [6,7]. 

Whilst usually muscle strains and sprains spontaneously heal, surgical intervention is the only established treatment for a complete tear at the MTJ; however, satisfactory long-term outcomes after surgery are limited due to adhesions and there is a high risk of re-rupture due to reduced mechanical properties [8]. A limited number of studies have been carried out on the MTJ and, therefore, questions associated with injuries and healing remain unanswered [9,10]. A description of the strain distribution at the MTJ would provide information on the mechanisms of injuries and requirements for ensuring a satisfactory repair, which is also associated with the ability of the MTJ to heal during rehabilitation. It has been demonstrated that the strain at the tendon close to the muscle is five times higher than in the midsection and the region of the tendon close to the bone [11]. Studies have reported that the MTJ exhibits the lowest stiffness in the muscle-tendon unit [12]. These findings indicate that the MTJ is the most susceptible region to strain changes in the muscle-tendon-bone unit and high stains in this region can lead to injuries. However, the 3D strain pattern at this junction when under a load has not been described.

The strain in the muscle-tendon unit in vivo has been measured; however, there is a large variation between the studies, potentially due to the size of the tissues [13,14,15,16,17]. Some authors reported that strain in the tendon was lower than that at the aponeurosis connected to the muscle [14,15,16], whist others have reported opposite findings [17,18] (Table 1). Studies have employed different techniques to visualize the tissue in order to investigate the mechanical behavior. The most common 3D-imaging technique for soft tissue in vivo strain measurement is ultrasound [19]. 

Higher resolution of the strain distribution in the muscle and tendon have previously been evaluated using digital image correlation (DIC) [20,21]. This technique evaluates strain on the surface of the tissues by analyzing sets of images of the specimen surface in undeformed and deformed states [22]. DIC has been used to investigate the strain on the surface of the Achilles tendon [21,23] and associated muscle [20]; however, this technique can only provide information on the surface strains.

Strain measurements of the muscle and tendon in 3D have been studied using finite element analysis (FEA) to predict deformation behavior [1,24,25,26,27]. Rehorn et al. reported that the stretch of the fiber in proximal MTJ before breaking was 1.64 times, which was higher than in muscle tissue (0.95 times) [25] Blemker et al. showed that shear strain was concentrated near the aponeurosis in human biceps brachii, which reached 2.4 ε, whereas some parts of muscles showed zero ε during low-load elbow flexion. In this study, the tissue was modeled as a fiber-reinforced composite with transversely isotropic material symmetry [27]. Cine phase contrast magnetic resonance imaging (MRI) has been used to investigate volumetric 3D strain of the human tibialis anterior during ankle plantarflexion–inversion [28].

Digital volume correlation (DVC) is a three-dimensional technique where the full-field strain distribution is derived from the measured displacement field, which is computed based on the differential variation in grey level intensity for 3D images of the material [29]. This technique is becoming widely used for biological tissues such as bone [29,30,31], tendon [32], and cartilage [33]. It has been combined with various imaging techniques such as X-ray computed tomography (XCT) [34], synchrotron tomography [35], and MRI [36].

Synchrotron imaging provides high beam spatial coherence and flux to image soft tissues with higher contrast and shorter imaging time than XCT [35,37]. The high flux of synchrotron allows for the capture of the images with high signal-to-noise ratio and high spatial resolution [38]. Phase contrast is often combined with synchrotron imaging due to its ability to image soft tissues without using contrast agents [39,40]. Synchrotron imaging has been reported to be an appropriate technique for phase contrast in order to achieve high accuracy soft tissue imaging [41] as well as DVC analysis [35]. However, the high flux of synchrotron imaging can cause radiation damage on soft tissues and therefore affect the accuracy of DVC [35]. 

High-resolution XCT has been previously combined with DVC to investigate the 3D full-field strain distribution on soft tissues [42]. One of the advantages of XCT is that it offers insights into the internal composite structure [43]. Soft tissues have low X-ray attenuation; therefore, to achieve sufficient contrast in the microstructure, techniques such as phase contrast and the use of staining agents have been employed [44]. XCT is often used to image the microstructure of soft tissues due to its ability to measure fiber alignment and volume fraction [45], and obtain morphological information such as shape, porosity, and roughness [46]. XCT can image specimens in both micro- and macro-level resolution, which is higher than the resolution of other techniques such as MRI (1–2 mm) and ultrasound (0.5 mm) [43,47,48]. Compared to MRI, XCT is more cost-effective and has shorter scan time [49]. Synchrotron has higher resolution and shorter image acquisition times than lab-based CT; however, it also has a higher beam current and flux of radiation, which can cause damage to soft tissues [37,50,51]. Phase contrast XCT is useful for soft tissue imaging [52], however, it requires a longer scan time than XCT without phase contrast to acquire sufficient number of projections [53], which can also damage specimens. A previous study on trabecular bone showed that 9-h XCT imaging did not cause any radiation damage on specimens [54]. Therefore, XCT was chosen as an imaging technique to be used for the strain measurement in this study.

The aim of this study is to utilize XCT imaging and DVC analysis to better understand the mechanical behavior of the MTJ related to the tissue microstructure. In this study, mouse Achilles MTJs were treated with phosphotungstic acid (PTA) to enhance the tissue contrast, and DVC was performed on these datasets to evaluate the mechanical behavior of the junction under a tensile load.

## 2. Materials and Methods

### 2.1. Specimen Preparation and Phosphotungstic Acid (PTA) Staining

Achilles tendons and gastrocnemius muscles were harvested from the right legs of adult wild-type male mice (10–12 weeks old, 31.2 ± 0.7 g). Six specimens were immersed in an increasing ethanol concentration of 25, 50, and 70% ethanol for 90 min each followed by 1% PTA (79690, Sigma-Aldrich, Burlington, MA, USA) in 70% ethanol for 72 h. They were then washed twice and immersed in phosphate-buffered saline (PBS) (P4417, Sigma-Aldrich, Burlington, MA, USA) for 1 h prior to mechanical testing or XCT scanning. Three of these specimens underwent mechanical testing and three specimens (referred to as S1, S2 and S3) were used for the XCT scanning and DVC analysis.

### 2.2. Tensile Testing Using Bose ElectroForce

In order to identify the elastic region of the tissues, mouse MTJs (*n* = 3) were tested under a tensile load using Bose ElectroForce 3200 (Bose ElectroForce, Framingham, MN, USA), which has a maximum load of 225 N. The load was measured as the specimens were loaded at a constant rate of 2 mm/min until the specimens failed. The tendon, bone, and MTJ were aligned so that the tensile force was applied along the length of the tendon-muscle unit. The specimens were prepared in the same way (stained using 1% PTA in 70% ethanol) as the in situ tensile testing described in Section 2.3 in order to determine the setting for in situ tensile testing. Specimens did not fail or undergo plastic deformation at 2.4 N load.

### 2.3. In Situ X-ray Computed Tomography (XCT) Mechanical Testing

In situ XCT imaging was achieved using the Versa 610 (Carl Zeiss Microscopy, Oberkochen, Germany) at 40 kV/3 W, with a 0.4× objective and 4 s exposure. One dataset took 3 h to obtain. In situ tensile testing was performed using the CT500 loading stage (Deben, Suffolk, UK), securing the top part of the muscle and the calcaneus part of the Achilles. To avoid slipping during mechanical testing the muscle was wrapped in sandpaper and glued with Loctite Superglue Precision (Loctite, Rocky Hill, CT, USA). To maintain tissue hydration during scanning, specimens were wrapped in gauze soaked in PBS and then wrapped in Parafilm (11762644, ThermoFisher Scientific, Waltham, MA, USA). The tendon, bone, and MTJ were aligned so that the tensile force was applied along the length of the tendon-muscle unit. 

During running, the load transmitted through the human Achilles tendon is between 6 and 8 times body weight (BW) [55,56,57]. In this study, the average weight of the male mice was 31 g, which translates into 2.4 N load (8 BW), which was applied to the specimens during in situ testing. In this study, an initial preload of 0.2 N was applied and two consecutive tomograms were acquired to calculate DVC strain uncertainties [58]. The specimens were then subjected to tensile deformation at a rate of 0.1 mm/min until 2.4 N load was achieved. All tests were carried out at room temperature. Following the initial tensile load, the specimens were then unloaded for 30 min, allowing stress relaxation before acquiring the last XCT dataset at 2.4 N using the same loading conditions. 

### 2.4. Image Postprocessing

The 3D datasets obtained by XCT after reconstruction (16-bit) were rigidly registered with a correlative metric in Avizo (Avizo 9.7, ThermoFisher Scientific, Waltham, MA, USA) against the 1st preload dataset. The volume was then cropped at 400 × 500 × 480 voxels (0.48 mm^3^) with the MTJ in the region of interest (ROI). Non-MTJ tissues were given a zero-intensity value in the grey scale by applying a mask to each dataset. The mask was applied by using an arithmetic operation between the original dataset and the binarized volume. This was followed by a median and unsharp filters to improve the tissue contrast and to remove the noise [59].

### 2.5. Digital Volume Correlation and Statistical Analysis

DVC (DaVis v10.0.5, LaVision Ltd., Gottingen, Germany) was performed between the first preload and loaded dataset to compute the 1st principal (ε_p1_), shear (γ), and von Mises strains (ε_VM_); a multi-pass scheme from 100 to 30 sub-volume voxel size with a 0% overlap was used, followed by a vector postprocessing, where the correlation coefficient was given a threshold of 0.6 [30,60]. Further details of the operating principles of DVC have been reported previously [34,61]. Error analysis with the 3 different specimens showed an average error value ± SD of 1519.11 ± 175.93 µε. The average value of strain in the ROI was obtained by the histogram function in Avizo. Tendon diameter and the volume loss between two preload scans were measured using Avizo. Two-way ANOVA was performed to calculate strain value and volume differences (*p* < 0.05) between the specimens. All statistical analysis was performed using GraphPad Prism 8.0.2 (La Jolla, Irvine, CA, USA).

### 2.6. Histology

Following testing, the MTJ was processed for histology. The specimens used for histology were (i) unstained MTJ without XCT imaging with a tensile load; (ii) PTA-stained MTJ without XCT imaging with a tensile load; (iii) PTA-stained MTJ with XCT imaging and without a tensile load; (iv) the PTA-stained MTJ specimens which were used for XCT imaging and DVC analysis (S1, S2 and S3). Specimens were fixed overnight using 4% paraformaldehyde (158127, Sigma-Aldrich, Burlington, MA, USA) and washed with PBS and then were dehydrated in 70%, 90%, and absolute ethanol for 30 min in each solution. They were then cleared in 3 changes of xylene (28973.328, VWR, PA, USA) for 9 h, before being transferred into paraffin wax (327204, Sigma-Aldrich, Burlington, MA, USA) overnight at 60 °C. They were embedded in paraffin wax. Eight-micrometer-thick sections were made using a Leica RM2235 rotary microtome (Leica Biosystems, Nussloch, Germany). The sections were stained with Harris hematoxylin (HHS32-1L, Sigma-Aldrich, Burlington, MA, USA) and eosin (HT110116, Sigma-Aldrich, Burlington, MA, USA) (H&E) before being mounted with DPX (#06522; Sigma-Aldrich, Burlington, MA, USA) and imaged using a Leica DMi1 microscope equipped with a Leica MC170 HD camera (Leica Microsystems, Wetzlar, Germany). After embedding, the specimens used for the DVC analysis were reimaged in the XCT so that the XCT dataset could be correlated to the histology. The area of the gap between the muscle fibers and tendon fascicles were measured using ImageJ in order to determine the level of damage in the tissues.

## 3. Results

### 3.1. Tensile Testing

Tensile testing of mouse MTJs was performed in order to determine the appropriate load to apply to the specimens for strain distribution analysis. The average ultimate tensile force of the three mouse specimens tested was 4.05 ± 0.45 N, with failure initiated by tearing, which was through the MTJ, and started at approximately 3 N.

**Figure 1:**
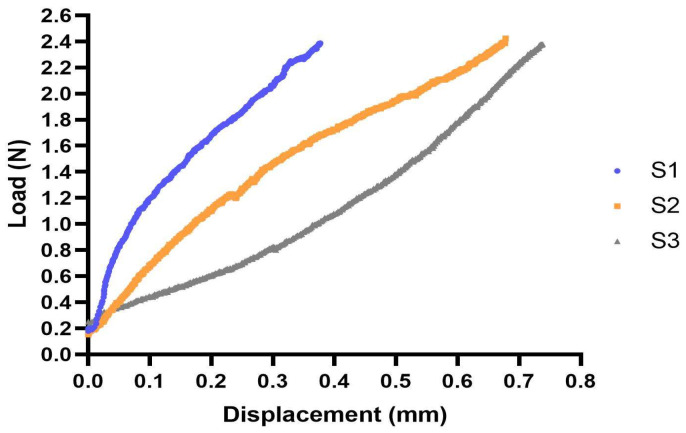
Load displacement curves of the three specimens under tensile loading in the elastic region. S1: blue, S2: orange, and S3: grey.

For the samples in the XTC, three sets of preload and loaded scans up to 2.4 N were acquired and a load displacement curve for each specimen was obtained (Figure 1). The average elongation of the specimens ± SD was 0.58 ± 0.18 mm. Notably, S1 exhibited less elongation compared to S2 and S3. Furthermore, S1 presented the widest tendon diameter (0.92 mm) in contrast to S2 and S3 (0.79 mm and 0.80 mm, respectively). This result showed that the tendon of S1 was stiffer than S2 and S3.

### 3.2. Strain Maps

**Figure 2:**
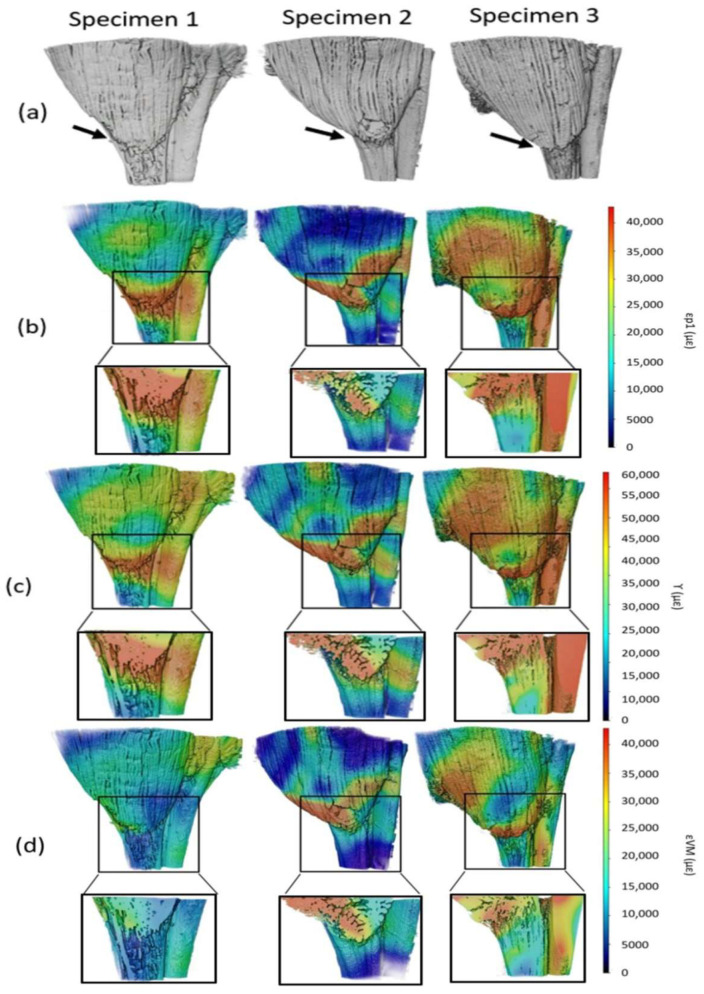
XCT-scanned mouse MTJs. (**a**) Proximal view; the muscle was in the top and the black arrows identified the MTJs in the middle of the images; (**b**) 1st principal strain; (**c**) shear strain; (**d**) von Mises strain for S1, S2 and S3 on the surface and in cross-sections of the MTJs. The cross-section through the MTJ was shown in the boxed region below the surface strains. The strain varied between 0 and 40,000 µε in (**a**,**c**) and 0 and 60,000 µε in (**b**) throughout the volume. The red area showed high strain and the purple/blue area showed low strain.

The XCT datasets showed the morphology of the mouse MTJ (Figure 2a). The first principal strain (ε_p1_), shear strain (γ), and von Mises strain (ε_VM_) maps were shown in the lateral view and in cross-section in Figure 2. High strain concentration was observed at the MTJ of each specimen for ε_p1_ (Figure 2b), γ (Figure 2c), and ε_VM_ (Figure 2d). All specimens showed similar strain distribution, with the highest strain concentration at the junction. The strain values in all specimens reached 120,000 µε, 160,000 µε, and 120,000 µε for ε_p1_, γ, and ε_VM_, respectively. DVC error analysis between two preload datasets showed that the average error value ± SD was 1519.11 ± 175.93 µε. The error values were less than 10% of the calculated strain, which showed that the level of error was acceptable. The peak strains of ε_p1_ and γ were observed in S3 and the peak strain of ε_VM_ was observed in S2. Muscle fascicles were not evenly strained, with some of the fascicles showing higher strain concentration (50,000 µε, 80,000 µε, and 60,000 µε for ε_p1_, γ, and ε_VM_, respectively), shown in red in the strain maps, whereas others showed low strain concentration, shown in purple or dark blue in Figure 2b–d. Notably, ε_p1_ showed elevated strain values at the MTJ, especially on the muscle side of the MTJ in S1 and S2, whereas, for S3, elevated values were also present in the tendon (Figure 2b). The shear strain demonstrated similar patterns to ε_p1_ (Figure 2c), reaching higher values of 40,000 με. Von Mises strain patterns resembled the other strains in the sense that the highest concentration was located at the junction; however, the absolute strain values varied among specimens (Figure 2d). S1 showed lower strain values throughout the tissue, where the strain value at the interface (35,000 µε) was 2–3 times lower than S2 and 3 (85,000 µε and 120,000 µε, respectively).

**Figure 3:**
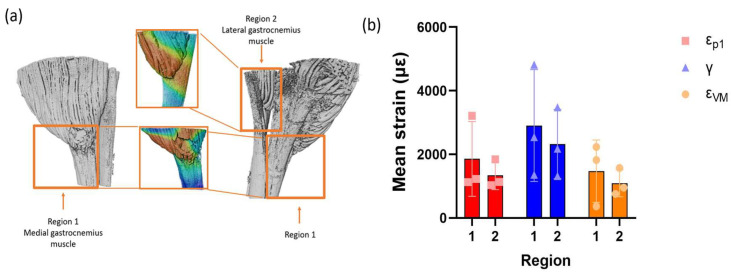
(**a**) Region selection from the overall scans of S2. R1 was selected from the medial part of the overall scans and R2 was selected from the lateral part of the images. (**b**) Average strain values of each type of strain in each region; red: 1st principal strain (με), blue: shear strain (με), and orange: von Mises strain (με). Data were presented as average ± SD (*n* = 3). Two-way ANOVA was used to calculate the significance between the regions. No significance was detected.

The interfaces between individual tendon fascicles and associated muscle were identified and the strain distribution in two different fascicles of the tendon was measured for each specimen. The anterior tendon fascicle within medial gastrocnemius muscle was named as region 1 (R1) and the posterior tendon fascicle within the lateral gastrocnemius muscle was named as region 2 (R2) (Figure 3a). The average value for each type of strain in each tendon fascicle was obtained (Figure 3b) and showed no significant difference between the two selected regions, although the mean strain value was 24.7 ± 3.7% lower in R2 than in R1 in each case (*n* = 3). 

**Figure 4:**
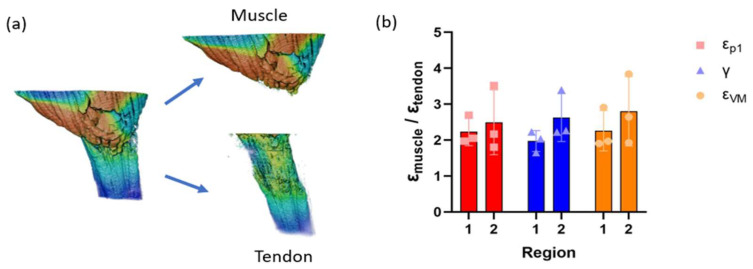
(**a**) Isolation of the muscle and tendon from the interface, R1. (**b**) Relative value of muscle strain at the interface compared to tendon; red: 1st principal strain (με), blue: shear strain (με), and orange: von Mises strain (με). Data were presented as average ± SD (*n* = 3). Two-way ANOVA was used to calculate the significance between the regions. No significance was detected.

The muscle and tendon at R1 and R2 from all the specimens were separated in order to measure the strain value of each tissue individually (Figure 4a). The relative value of muscle strain compared to tendon strain (ε_muscle_/ε_tendon_) was obtained at each interface and the average values of all three specimens were shown in Figure 4b. The ε_muscle_/ε_tendon_ ratio was above 1.8 in every specimen and region for all three strains, indicating that the strain concentration in the muscle was higher than in the tendon. No significant difference was detected between the regions. 

### 3.3. Volume Strain Correlation

**Figure 5:**
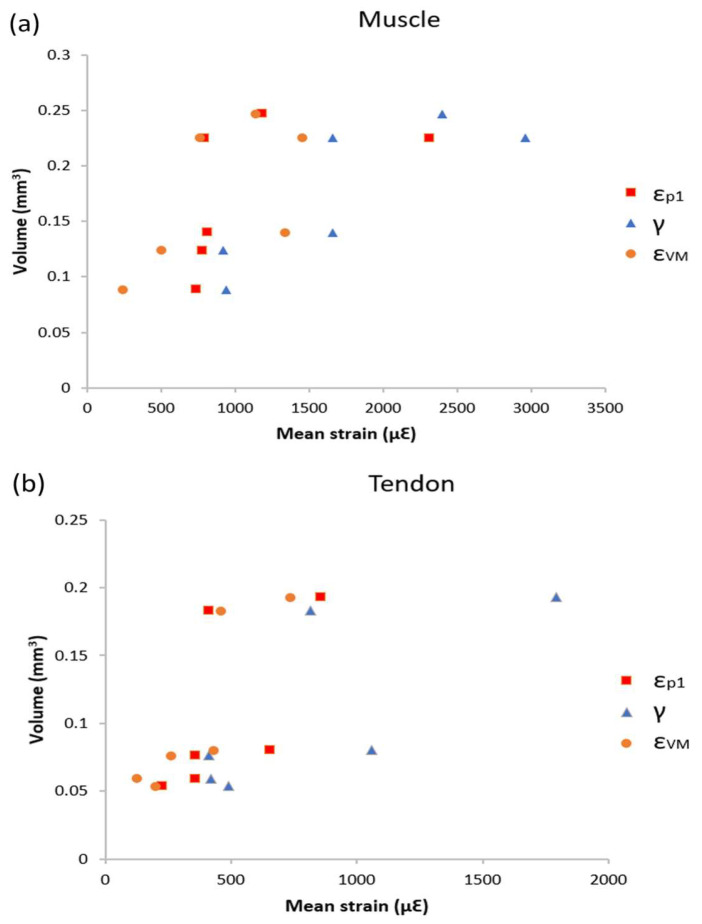
Scatter plots of the relationship between volume of (**a**) the muscle, (**b**) the tendon and strain (*n* = 6, R1 and 2 from all specimens); red: 1st principal strain (με), blue: shear strain (με), and orange: von Mises strain (με).

The volume of muscle and tendon fascicle of all specimens at R1 and 2 were obtained. Figure 5 showed the volume strain correlation at the muscle (Figure 5a) and the tendon (Figure 5b). R-squared values were calculated for each strain type. The average R-squared values ± SD of three strain types of the MTJ were 0.458 ± 0.165 and 0.562 ± 0.150, respectively. This result showed that there was moderate correlation between the volume of tissues and the strain values.

### 3.4. Histology

**Figure 6:**
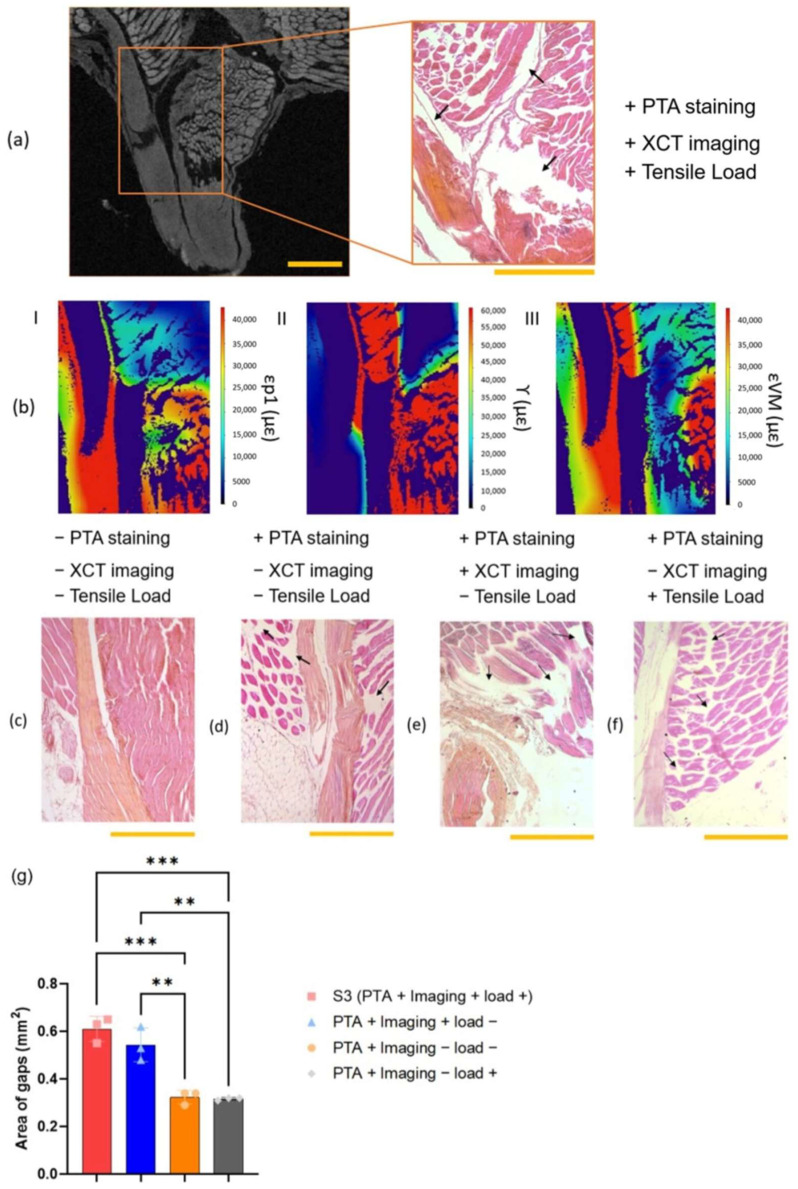
(**a**) A histology image of S3 and XCT image correlated to the histology image; the scale bar was 500 µm. (**b**) Cross-section of strain maps that were correlated to the histology image (I for εp1, II for γ, and III for ε_VM_). (**c**) A histology image of unstained mouse MTJ. (**d**) A histology image of PTA-stained mouse MTJ without XCT imaging and a tensile load. (**e**) A histology image of PTA-stained mouse MTJ with XCT imaging and without a tensile load. (**f**) A histology image of PTA-stained mouse MTJ without XCT imaging with a tensile load. Black arrows showed gaps in the tissues created by dehydration. The scale bar was 500 µm. (**g**) Average area of gaps between muscle and tendon fibers (*n* = 3). One-way ANOVA was used to calculate the significance between the specimens (** *p* < 0.01 and *** *p* < 0.001).

Histology was performed on unstained mouse MTJ, PTA-stained mouse MTJ with and without XCT imaging, as well as XCT-imaged MTJ with and without tensile loading. This allowed for evaluation of the effects of the PTA staining, XCT imaging, and a tensile load on the tissues. The muscle tissues were stained a darker pink than the tendon tissues due to the low cell population in the tendon. Compared to the unstained specimen (Figure 6c), PTA-stained specimens (Figure 6a, d–f) showed a number of artefacts caused by staining with PTA and/or exposure in the XCT (identified by black arrows in Figure 6c–f). H&E staining was performed on S3 after it had been stained with PTA, scanned, and loaded under tension in the XCT (Figure 6a). Figure 6b showed the distribution of each type of strain (ε_p1_, γ, and ε_VM_) in the corresponding area of the histological section in Figure 6a. The highest value for each of the different strains was observed at the interface between the muscle and the tendon (110,000 με, 150,000 με, and 80,000 με for ε_p1_, γ, and ε_VM_, respectively). 

In order to investigate the effects of dehydration on XCT imaging and loaded tissues, the region between the muscle and tendon fibers in S3, PTA-stained MTJ with XCT imaging without a tensile load, PTA-stained MTJ without XCT imaging with a tensile load, and PTA-stained MTJ without XCT imaging with a tensile load was measured (*n* = 3). The histology images from S3 were representative and were reported here. The average area values ± SD were 0.61 ± 0.043 mm^2^ (PTA stained/XCT), 0.55 ± 0.053 mm^2^ (PTA stained/XCT/loaded), 0.32 ± 0.024 mm^2^ (PTA stained/loaded), and 0.32 ± 0.005 mm^2^ (PTA stained) (Figure 6g). There was a significant difference in the area when XCT-imaged MTJ and non-imaged MTJ were compared, indicating that the methods used for XCT imaging created artefacts compared to normal MTJ; however, there was no statistical difference between loaded MTJ and unloaded MTJ.

The shrinkage of specimens caused by XCT imaging was detected by calculating the volume loss between two preload scans. The average percentage ± SD of the lost volume in the second scan compared to the first scan was 4.93 ± 1.71% (*n* = 3).

## 4. Discussion

XCT combined with DVC analysis enabled the 3D strain distribution in the mouse MTJ to be measured. High strain was observed in the junction (Figure 2), which can be attributed to the load transfer between the two different tissues (muscle and tendon). High strain in this region has also been detected in previous studies that investigated the rat tibialis anterior muscle-tendon-bone unit using an optical force transducer and two uniaxial servomotors producing a constant tensile load [11]. In our study, DVC allowed for a full-field analysis of the strain throughout the volume of the tissue as well as on the surface of the tissue (Figure 2). Strain was concentrated at the MTJ and was 3–12 times and 2–10 times more than that in the adjacent tendon and muscle, respectively. As shown in this study and reported previously, the strain distribution in muscle fascicles varies and this has been shown to be dependent on their length and curvature [27,62]. The results presented in our study help to better understand the 3D MTJ deformation and how strain is distributed from the muscle to the tendon, predicting the site of failure under tensile load.

The high variability in the strain distribution between the three different samples was to be expected because of the differences in the morphology, size of the tissues, and fascicles in specimens. The dependence of strain on tissue volume has been reported previously [63,64,65]. The tensile load displacement curves of the three specimens investigated showed shorter elongation of S1 compared to S2 and S3, and this could have been due to the different thicknesses of the tendons. S1 had the widest tendon diameter (0.92 mm) compared to S2 and S3 (0.79 mm and 0.80 mm, respectively), and as expected and reported previously [64], the tissue size and individual variation affected mechanical properties [66]. The load displacement curves indicated that all three specimens were tested in the elastic region of the muscle-tendon unit. Furthermore, the morphology of specimens with and without a tensile load were analyzed to demonstrate the effects of loading on the integrity of the MTJ. The area between the muscle and tendon at the junction showed no significant difference between the loaded specimens and unloaded specimens (Figure 6g), again indicating that the samples were deformed elastically.

The high-resolution lab-based XCT images (5 µm voxel size) in this study showed the morphology and orientation of the muscle and tendon at the fiber and fascicle level and showed the strain distribution at the interface was relatively uniform, with decreasing strain values from the MTJ towards the tendon. The strain distribution at the interface between an individual tendon fascicle and connected muscle fibers was analyzed and the average value of each strain was shown to be not statistically different. This result suggested that the strain distribution at the interface of individual tendon fascicles was uniform under a tensile load. Similarly, the transfer of strain from the muscle to the tendon was evenly distributed across the MTJ. Out of the reported strains, shear strain reached the highest value (160,000 με) and this may be due to the sliding of the fascicles associated with their fibrous interconnectivity, which often happens in a plane that is not completely orthogonal to the direction of the tensile load as suggested previously by others [67,68]. Although the specimens were tested within their apparent elastic region, high von Mises strain values were present in the MTJ, which could indicate that the tissue was under yield locally. The human Achilles tendon yield strain has been reported to be between 40,000 and 960,000 με during running [69], however, information on the yield strain at the MTJ is limited. The results here showed that, for a load of 2.4 N for the mice Achilles muscle-tendon unit, which was equivalent to eight times the body weight [57]. The von Mises strain reached 120,000 με locally and yield of the MTJ was predicted. S1 had lower von Mises strain values throughout the tissue compared to S2 and S3. This may be caused by the thicker tendon diameter (0.92 mm) compared with the thickness of S2 and S3 (0.79 mm and 0.80 mm, respectively). Boyer et al. analyzed the correlation between tendon size and mechanical properties in situ by using pull-through tests of the single double-stranded transverse suture and reported that there was a positive correlation between the tendon width and yield strength [70].

Contradicting results of strain measurements of the muscle and tendon in vivo have been reported in the literature (Table 1), where some reported that the muscle had higher strain than the tendon under a tensile load, while others showed the opposite [14,15,16,17,18]. Specifically, Maganaris et al. measured strain in human muscle-tendon unit using ultrasound under stimulated contraction and reported that strain in the tendon was 0.8 to 2.5%, which was lower than that in aponeurosis (2.1 to 7%) [14]. Finni et al. also demonstrated in vivo strain measurement in human muscle-tendon unit using MRI under maximal voluntary contraction; however, they reported that the strain in tendon (2.8 to 4.7%) was higher than in the aponeurosis (1.2 to 2.2%) [17]. In this study, the ε_muscle_/ε_tendon_ values at the interface between the muscle and tendon were greater than 1.8 for ε_p1_, γ, and ε_VM_. Our study demonstrated that the mean strain values of the muscle were consistently higher than those of the tendon under a tensile load, even though the strain distribution in the muscle fibers was not uniform. Therefore, the variation in previous studies may be due to the unevenness of the strain distribution in the muscle.

Correlations between tissue volume and strain showed that the R^2^ values ± SD for the muscle was 0.458 ± 0.165 and, for the tendon, the average R^2^ value was 0.562 ± 0.150 (Figure 5), suggesting that the volume of muscle and tendon had moderate correlation with strain values [71]. It was also reported previously that the bigger skeletal muscle showed an increase in local strain [66]. This result suggested that one of the reasons for the high variability in MTJ strain in these specimens can attributed to the differences between the size of the muscle-tendon unit.

Histology showed micro fracture at the MTJ junction in the images for S3 (Figure 6a), which could be attributed to the effect the staining and XCT imaging had on the mechanical properties of biological tissues. However, the strain maps showed that the load was transferred from the muscle to the tendon despite this discontinuity.

A limitation of this study was the use of PTA in 70% ethanol for staining the tissue, which was required to increase contrast in laboratory XCT. Dehydration caused a significant change in morphology and mechanical properties in the muscle and tendon [72,73]. There were two main factors in our methodology that could contribute to inaccurate absolute values, and these factors were associated with a combination of PTA staining and damage associated with the radiation of the XCT scan. Histological analysis was performed to determine the effect of the staining and scanning process had on the tissue structure of the specimens. The shrinkage observed in Figure 6d compared to that in Figure 6c suggested that the PTA staining caused the shrinkage, and this was due to the staining process using PTA dissolved in 70% ethanol. The effect of dehydration on soft tissues using PTA was an important observation because this would affect the absolute strain values. Although shrinkage of PTA-stained soft tissues has previously been demonstrated in the literature and it has been shown to affect tissue mechanical properties, PTA has been reported to have a more limited effect on tissue dehydration compared to other contrast agents such as iodine [74]. Using our PTA staining protocol, the results presented illustrated the mechanical behavior across the MTJ. Even larger gaps between the muscle fibers and tendon fascicles were detected on the PTA-stained specimens after XCT imaging than on the stained specimen without XCT imaging (Figure 6g), and this suggested that the imaging process could have affected the tissue further through dehydration or radiation damage [54]. Even though XCT has this limitation, it has advantages over other imaging techniques that have been used to analyze strain distribution. For example, MRI is more expensive, and has longer scan times, and lower resolution [43,49]. Synchrotron has higher resolution than XCT; however, it also has higher beam current [37] and has been reported to damage biological macromolecules, which affects the morphology and mechanical properties of the specimens [75,76]. It has been reported previously that the temperature inside the XCT increases by only 2 °C after 3-h XCT imaging [54]. Therefore, the damage on the specimens through the XCT imaging process may be less than the other imaging techniques.

In order to avoid specimen dehydration, a new staining method for the soft tissues that does not use a dehydrating solvent needs to be developed. It has been reported that PTA can be prepared in water instead of ethanol to avoid specimen shrinkage [74], however, our preliminary use of saline did not result in penetration of the PTA throughout the mouse tissue even after 6 days of incubation at 4 °C. Therefore, 70% ethanol was used as the staining solvent in this study. Imaging the specimens in hydration liquid is another way to avoid specimen dehydration; however, a liquid cell for in situ tensile testing would make the distance between the source and the specimen larger, which results in lower resolution. Therefore, the specimens were covered by gauze immersed in PBS in order to maintain a wet environment and minimize the dehydration of the specimens. Future work should include the investigation of tissue staining and using different imaging techniques to minimize the damage on the tissues. 

Phase contrast imaging in the XCT does not require the soft tissues to be stained and potentially could overcome the staining issue [77]. However, the application of phase contrast imaging has been limited due to the restricted number of suitable X-ray sources [78]. In addition, although phase contrast imaging seems to be a promising approach for soft tissue imaging for lab-based CT machines, it requires a long scan time to acquire a sufficient number of projections, which is limiting and can also cause the dehydration of the specimens [53]. Synchrotron imaging has a shorter imaging time than XCT [35,37], therefore, combining phase contrast synchrotron imaging may solve the imaging time issue and achieve less damage on the specimens. However, synchrotron imaging can induce more specimen damage due to its high beam current and flux of radiation compared to XCT [37,50,51]. Further investigation is needed to measure specimen damage using different techniques. Reducing the number of projections also reduces the imaging time; however, this can cause poor reconstruction quality [79]. Therefore, future work includes the optimization of imaging protocol such as comparison between XCT imaging and synchrotron imaging with phase contrast.

Another limitation was the small number of specimens (*n* = 3) used for DVC analysis. However, it should be noted that DVC analysis is a technique to qualitatively assess local strain distribution in 3D [30]. Although the number of the specimens used was small, the results shown in this study, comparing the strain values across individual samples, showed that strain distribution between the muscle and tendon was similar in all specimens. 

## 5. Conclusions

This study explored the 3D full-field strain distribution in the MTJ under tensile loading for the first time. High strain concentration was measured at the junction and allows for better understanding of MTJ deformation, strain distribution, and injury in this region. These results may be useful to predict where tears occur, providing information that would allow more effective treatments of tears at the MTJ. Furthermore, the results showed that the strain at the muscle was higher than the one at the tendon under a tensile load. The staining and imaging method should be improved to avoid specimen dehydration by developing a new staining protocol without a dehydrating solvent.

## Figures and Tables

**Table 1:** Strain values of tendons and their aponeurosis connected to muscles from published studies.

Strain in Tendon	Strain in Aponeurosis	Load Applied	Subject	Imaging Technique	Reference
0.8 to 2.5%	2.1 to 7%	five series of stimulated contractions at 20, 40, 60, 80, and 100% of maximum isometric dorsiflexion moment	human tibialis anterior tendon	Ultrasound	[14]
4.72 ± 1.85%	5.12 ± 2.07%	isometric maximal voluntary contractions	human Achilles tendon	Ultrasound	[15]
1.95 ± 1.49%	7.95 ± 7.47%	passive loading to a tension equal to maximum tetanic contraction	frog semitendinosis tenodn	He-Ne laser	[16]
2.8% and 4.7% respectively	1.2% and 2.2% respectively	20 and 40% of maximal voluntary contraction	human Achilles tendon	Magnetic resonance scanner	[17]
8.0 ± 1.2%	1.4 ± 0.4%	voluntary isometric contraction	human Achilles tendon	Ultrasound	[18]

## Data Availability

Data are contained within the article.
